# The role and attributes of social networks in the provision of support to women after stillbirth: experiences from Uganda

**DOI:** 10.1186/s12905-021-01498-9

**Published:** 2021-10-06

**Authors:** Eric Ssegujja, Yusuf Mulumba, Sally Guttmacher, Michelle Andipatin

**Affiliations:** 1 Makerere University School of Public Health, Kampala, Uganda; 2 School of Public Health, University of the Western Cape, Cape Town, South Africa; 3 Uganda Cancer Institute, Mulago National Referral Hospital, Kampala, Uganda; 4 School of Global Public Health, New York University, New York, NY USA; 5 Department of Psychology, University of the Western Cape, Cape Town, South Africa

**Keywords:** Stillbirth, Social support, Social network

## Abstract

**Introduction:**

Communities exert stigma on mothers after stillbirth despite their potential to offer social support to the grieving family. Maternal healthcare-seeking behaviors are socially reinforced rendering a social network approach vital in understanding support dynamics which when utilized can improve community response to mothers experiencing stillbirth. However, the form and direction of social support for women when in need is not clear. The study explored the role and attributes of women’s social networks in the provision of support to mothers who have experienced a stillbirth in Uganda.

**Methods:**

An exploratory cross-sectional study design adopting a social network approach was conducted. Data collection following established procedures was conducted on a convenient sample of 17 mothers who had experienced a stillbirth six months before the study. Frequencies and bivariate analysis were conducted to determine the factors influencing the provision of social support from 293 network members elicited during the alter generation. We then performed a Poisson regression on each of the social support forms and the explanatory variables. Network structure variables were calculated using UCINET version 6 while Netdraw facilitated the visualization of networks.

**Results:**

Overall, social support was available from all network relations mentioned by the respondents. No major variations were observed between the two time periods during pregnancy and following a stillbirth. The most common support received was in form of intangible support such as emotional and information support, mainly from females who were married and from the naturally occurring networks such as family and friends. We also observed that social support followed patterns of network relational characteristics including trust, frequency of contact and alters counted on for support more likely to provide the same.

**Conclusions:**

A great potential for social support exists within women’s social networks to help address stillbirth risk factors during pregnancy and cope after experiencing the same. Alter characteristics like being female, married, and from naturally occurring networks together with relational characteristics such as trust, frequency of contact, and count on alter for support were predictors of eventual social support. Interventions aiming at addressing stillbirth risks at the community level ought to harness these network characteristics for benefits to the mothers.

**Supplementary Information:**

The online version contains supplementary material available at 10.1186/s12905-021-01498-9.

## Background

Social support to mothers during pregnancy is associated with a general sense of psychological wellbeing, self-worth and enables women to access resources for maternal health services which all contribute to improved quality of life for the mother-baby dyad [1]. Similarly, a display of depressive symptoms and anxiety postpartum has been associated with a lack of social support from the social environment [2, 3] with a lack of emotional and information support as the leading contributor for postpartum anxiety [3]. Social support is a modifiable risk factor and is measured as part of a comprehensive maternal health care assessment. It refers to the perceived and enacted care one receives from being part of a supportive social network [4] while social capital is the actual value embedded within the social relations that demonstrates the quality and quantity of such relationships. It manifests through the connections among network actors who demonstrate care by providing the different types of support to mothers in need [5]. At the community level where the relationships are embedded, the support has the potential to aid pregnant women in seeking the required care to avert a potential stillbirth risks and enhance adjustment to cope with life after for those that have experienced one.

Two mechanisms for social network influence through which social support manifests exist; connection and contagion. They trigger social capital among network actors to provide social support to a member in need. Connection refers to the individual’s standing within the wider network that attracts social support because of their position while contagion is the spread of perceived and enacted reciprocal behaviors to offer social support to members in need [6]. The network effect hypothesis akin to contagion postulates that similarities in lifestyle and health behaviors, emotions, cultural norms among individuals are a product of diffusion and influence in networks while the self-selection hypothesis synonymous with connection suggests that the ties among members are driven by pre-disposing beliefs and attitudes which influence the formation and sustenance of the networks [7]. Interventions utilizing network approaches to strengthen or build capacities within existing community relationships and connections are known to reinforce social support and are more sustainable compared to ones that either introduce new nodes or links to existing social networks due to their over-reliance on external social support [8].

On the one hand, community-level interventions targeting improved social support for better maternal and child health outcomes have deployed social network approaches in varying ways [8]. Strengthening existing networks through the identification of most influential persons by nominations or mathematical algorithm to engage them for behavioral change impact was behind the creation of mentor mothers to support Prevention of Mother to Child Transmission (PMTCT) programs [9]. Similarly, the involvement of male partners and mothers’ in-laws in maternal health especially in rural areas where such traditional norms and beliefs are held in high regard was intended to avoid unnecessary community delays due to maternal healthcare seeking decision-making dynamics [10–12]. Additionally, other interventions have used induction approaches where peer to peer interaction is stimulated between links of existing networks as witnessed when group antenatal care (ANC) counseling sessions [13, 14] and women savings groups are initiated for birth preparedness plans [15, 16].

On the other hand, the segmentation approach to network interventions seeks to change a group of people at the same time for improved maternal health behaviors. The inclusion of maternal health and Family Planning services onto the package delivered by Community health workers on top of managing childhood illnesses was specifically intended to infiltrate pregnant women’s networks with reliable maternal-related information and services [17]. Relatedly, programs that alter pregnant women’s social networks such as a ban on traditional birth attendants in Uganda and discouragement of the same thereof was intended to eliminate a critical network node characterized by community delays and late referrals in a bid to improve facility deliveries supervised by skilled birth attendants [18]. Similarly, the inclusion of motorcycle riders through a transport voucher scheme in Eastern Uganda served to reduce redundancy while increasing communication and access to community resources to improve the referral system for improved maternal health outcomes [19].

Global campaigns to address stillbirth were embraced at the national level with Uganda prioritizing interventions to address fresh stillbirth through health systems strengthening. The country registered a steady decline in fresh stillbirth from 16/1000 in 2013 to 9/1000 by 2019 ahead of the Every Newborn Action Plan(ENAP) and Health Sector Development Plan (HSDP) targets [20, 21]. While a number of interventions have focused at a health systems-level, more remains to be seen at the community level [22]. The strategy for a community intervention at scale with support from the World Bank’s Global Financing Facility (GFF) is yet to be rolled out. This trajectory falls short of achieving the global objective to reduce stillbirth [23, 24] especially in Sub-Saharan Africa where many community cases still occur coupled with late presentation for ANC with low completion rates [22]. It is then that interventions targeting social support to facilitate linkage into care promise a viable option to contribute to addressing the stillbirth burden in low resource settings. For international comparisons, WHO defines stillbirth as loss of a foetus after 28 weeks of gestation [23, 24]. This is the same definition that was adopted for Uganda.

For stillbirth prevention, a great deal of research into the community factors has focused on stigma from the community members to mothers and families experiencing a stillbirth [25, 26]. This masks the potential role that communities can play as active partners in addressing the problem. Interventions with potential optimal effect on stillbirth reduction are those that respond to equity in reaching the poor and marginalized as well as those dealing with behavioral practices [27]. Access to the right maternal health information can redirect mothers to appropriate service providers while the provision of transport can lead to a timely referral. The major challenge however remains the inability to tap into social support from networks to avert stillbirth.

This study aimed to explore the role and attributes of social networks in providing support to women who have experienced a stillbirth in Mukono district of Uganda. Elsewhere; kinship, trust, education, age, wealth, neighborhood context have been shown to influence social support for maternal health care service seeking and access [28–30]. Within Uganda, studies have demonstrated the crucial role of social support in helping patients access HIV services and act as agents for prevention [31, 32]. None has studied the role of women's social networks in the provision of social support before and after experiencing a stillbirth. The expected outcome of this study was to document the nature of available social support within existing networks for women experiencing a stillbirth and to characterize providers and recipients as well as the networks they belonged to inform how best these could be strengthened for community-level stillbirth prevention strategies.

## Methods

### Design and study setting

This was an exploratory cross-sectional study adopting a social network approach as part of a larger mixed-methods study conducted in Mukono district located in central Uganda with a high fertility rate. It has characteristics of a pluralistic health system with the public, private not for profit and private for-profit health facilities offering maternal and child health services. There are a total of 51 health facilities of which only four are at Health Centre IV level and above offering Comprehensive Emergency Obstetric care services. Some mothers accessing maternal health services at the health facilities come from the neighboring districts due to central location, proximity to the city, and access to the great east Africa highway; a major transport route in the country and region. Details of the study setting have been described elsewhere [33].

### Participant’s characteristics

Enrolled participants in this study included women in their reproductive age (18 years or older) with eligibility criteria of having experienced a stillbirth within six months before the study and consented to participate. They had delivered the index pregnancy in one of the health facilities in the district specifically focusing on Health center III and above because they offer emergency obstetric care services. Exclusion criteria included not being available for interview during the study period and those mothers who had delivered within one month prior to the study. The results reported here are from 17 respondents out of the 20 that were targeted and 23 who were approached.

### Description of the processes

Data were collected between January and May 2019 where a convenient sampling technique was applied to access potential respondents who were identified from facility records and health worker’s recollections. They were approached by the health workers from the maternity unit who were first oriented about the study and supported to gain confidence and clarity while explaining the objectives and processes to potential respondents. Health workers would first speak to the potential participant and inform them about the study and thereafter would request them if they were willing to participate after elaborating the study objectives. A study team member verified information with health providers to ascertain eligibility before contacting the mothers. Those that agreed to participate were then approached by the study team member using the information provided via mobile telephone. Thereafter a convenient place and time for the interview would be agreed upon with the potential respondent. Interviews were face-to-face interviewer-administered whereby on the day of the interview, the objectives and procedures to be followed would be repeated for the participant and consent obtained. Although the study did not set out to collect data on refusal to participate, it later emerged that three of the potential respondents that had been approached declined to participate and at that point, no further contact was made.

The tool used was developed by the first author based on literature and standard procedures for conducting social network interviews (attached here as Additional file 1). It contained five sections; section one elicited information on social demographics, household characteristics, maternal health, and obstetric history characteristics collected from each respondent through self-report. Section two included the name generator where an egocentric network approach was used to guide respondents to recall at least fifteen to twenty of their social network members. The criteria included giving names of those people they recall to have had contact or interacted with during pregnancy and after experiencing a stillbirth. This followed established processes for conducting social network interviews [34–36].

To assess network composition, section three asked respondents to provide information about each of the network members they had earlier listed which included demographic data such as age, education, gender, marital status, and relationship type. The level of trust, emotional closeness with network members, frequency of contact, and whether that particular network member could be relied on for support when in need. They were assessed on a three-point Likert scale including “Not at all, a little bit or Very much. The frequency of contact with a network member was assessed on a five-point Likert scale which reflected “1 = never, 2 = once a month, 3 = once a week, 4 = several times a week, and 5 = about every day”.

Section four covered the different aspects of social support explored between the respondent and network members. Specifically, following guidance from literature [28, 37], social support was conceptualized as consisting of five types including; financial, information, material, emotional, and instrumental support. The questions were repeated for each of the categories asking if respondents had sought or received support from the network member during pregnancy and after experiencing a stillbirth. The responses were on a three-point Likert scale; [1] “Not at all, (2) A little bit, and (3) Very much”. The last section (five) assessed the network structure where participants reported if a particular network member knew the other alters mentioned with response options including “Yes, No or don’t know. They were also asked about the nature of the relationship with network members in terms of Trust and count on alter for support which was assessed on a three-point Likert scale; (1) “Not at all, (2) a little bit or (3) Very Much”. Emotional closeness was assessed on a three-point Likert scale with (1) Not at all, (2) somewhat, (3) very close. Frequency of contact was assessed on a five-point Likert scale with (1) once in six months, (2) once in three months, (3) Once in a month, (4) once a week and (5) about every day.

### Definitions

*Emotional support:* behaviors that foster a feeling of comfort which leads to a person to believe that they are being admired, respected, loved, and that others are available to provide care and safety.

*Information support:* Knowledge, advice, or information that helps an individual to understand their world and adapt to the change that comes with it.

*Instrumental support:* the help from other people in terms of activities that the ego is unable to perform or for which others are required to help solve a problem.[help with household chores, accompany to the hospital] we separate instrumental from material as one refers to services while the other refers to tangible goods.

*Financial support: *assistance in terms of money to a mother to help her buy a good or facilitate a service.

*Material support:* This refers to tangible goods received by women to help solve a particular problem.

### Data analysis

Because the study adopted an exploratory approach, the results presented use descriptive statistics to characterize the study sample and network alters who were the primary providers of social support before and after experiencing a stillbirth. Descriptive analysis was conducted for the respondent and alters characteristics using frequencies and proportions. The prevalence of social support was calculated using frequencies, proportions, and chi-square tests with 95% confidence intervals. Alter characteristics and network relational characteristics reflecting qualities were considered explanatory variables. A bivariate analysis was conducted on both the alter and network characteristics to explore the association both during pregnancy and after experiencing a stillbirth. We then performed a Poisson regression on each of the social support types and the explanatory variables. Statistical analyses were conducted using Stata software with a significant level set at *p* < 0.005 while UCINET version 6 was used to calculate the social network structural measures for each of the respondents. Network graphs were produced using Netdraw.

## Results

The data presented are from 17 respondents although twenty had initially participated in the study, three cases were removed at analysis because they provided fewer alter during the name generation exercise.

### Respondent characteristics

Overall, respondents reported the mean age at first pregnancy as 20.5 years (range 14–30) with 65% reporting a parity of three or more at the time of the interview. Close to a quarter of the respondents (24%) indicated a history of a negative pregnancy outcome such as a stillbirth prior to the index pregnancy. Half of the index pregnancies (52%) that resulted in the stillbirth were referrals-in with the first point of care-seeking being mainly the lower-level public health facilities (44.4%) and private clinics (44.4%). Very few (16.7%) reported using any family planning method at the time with more than half (64.7%) reporting intentions to have another child within the next one to three years.

According to respondents’ demographics, the mean age was 29.4 years (range 21–41), the majority (41%) had some secondary level education with many of whom (88%) being Christians. The majority (83.3%) were married with more than half (59%) living with four members or less in the households and staying in a nuclear family arrangement (65%). Details are presented in Table 1.

**Table 1:** Respondents demographics (*N* = 17)

Respondent characteristics	*N*	Percentage	Mean	Range
Age			29.4	21–41
*Education*				
No education	1	6		
Primary	4	24		
Some secondary	7	41		
Completed secondary	5	29		
*Religious affiliation*				
Christian	15	88		
Muslim	2	12		
Marital status (married)	14	83.3		
Mean age at first pregnancy			20.5	14–30
Parity (3 and over)	11	65		
History of a miscarriage/stillbirth (yes)	4	24		
*Stillbirth pregnancy order*				
First	3	17.6		
Second	2	11.7		
Third	2	11.7		
Forth	3	17.6		
Fifth	5	29.4		
Sixth	2	11.7		
*Place of SB delivery*				
Facility	8	47		
Referral-in	9	52		
*First point of care-seeking*				
TBA	1	11.1		
Clinic	4	44.4		
Lower-level health facility	4	44.4		
Current use of family planning (yes)	3	16.7		
Intend to have more children (yes)	11	64.7		1–4
When intend to have a child			1 year	1–3 years
People living in the HH (four and below)	10	59		
Family type (Nuclear)	11	65		

### Network members (alter) characteristics

Network member characteristics reflected diversity where 70.6% were females, the majority (64.8%) with some secondary level education or more, and more than half (67.6%) being married. By composition, more than half (58.4%) were from naturally occurring network members of which 62.6% were family members. The majority (80.9%) of the alters were trusted by the respondents while 78.5% were in frequent contact with the respondent and 72% were emotionally close to the respondent. 66.6% of the alters would be counted on to offer support with 65.5% maintaining or improving their relationship with the respondent following a stillbirth. Details are presented in Table 2.

**Table 2:** Alter characteristics (*N* = 293)

Alter characteristics	Frequency	Percentage	Range
Age (26 >)	249	85	
Gender (female)	207	70.6	
*Education*			
No educ-primary	103	35.2	
Secondary and above	190	64.8	
Marital status (married)	198	67.6	
*Network member type*			
Naturally occurring network	171	58.4	
Community role network	122	41.6	
*Naturally occurring network member type*	*N* = 171		
Spouse	16	9.1	
Family	107	62.6	
Friend	48	28.1	
Trust (yes)	237	80.9	
Frequency of contact (frequent)	230	78.5	
Emotional closeness (close)	211	72	
Alter knows about SB incident (yes)	287	98	
*How alter got to know*			
Told by respondent	80	27.3	
Knew by themselves	69	23.5	
Through network members	143	48.8	
Through other means	1	.3	
*Relationship with alter after SB incident*			
As before or better	192	65.5	
Worse than before	101	34.5	
*Count on network member for support*			
Yes	195	66.6	
no	98	33.4	
Average network size	16		

## Prevalence of social support among network members

**Fig. 1:**
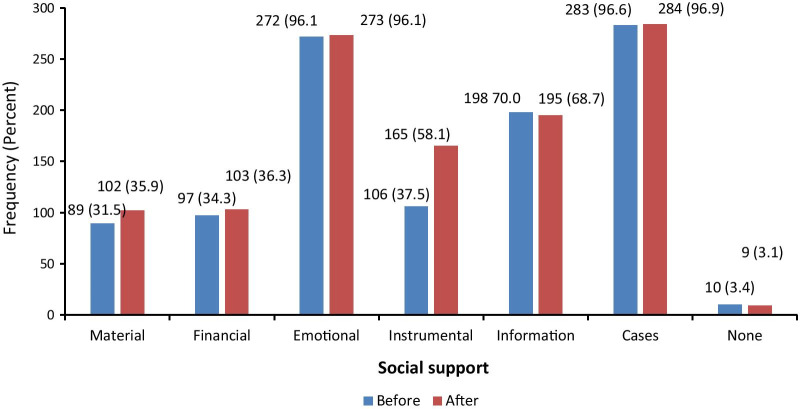
Prevalence of social support (during pregnancy and after experiencing a stillbirth)

Overall results revealed that emotional support was the most prevalent among network members both during pregnancy and after experiencing a stillbirth as reflected in Table 3. Results further show that there were no differences between emotional support received during pregnancy (96.1% CI 89.23–95.29) and after experiencing a stillbirth (96.1% CI 89.63–95.57). Material support was the least offered from network members both during pregnancy (31.5% CI 25.35–35.92) and when respondents experienced a stillbirth (35.9 CI 29.54–40.48). Details of the visualized results are reflected in Fig. 1.

**Table 3:** Bivariate association of social support with alter characteristics

Variable	Total	Material	*P *value	Financial	*P *value	Emotional	*P *value	Instrumental	*P *value	Information	*P *value
*During pregnancy*											
Age group in years											
Less than 18	9 (3%)	0 (0%)	0.199	0 (0%)	0.082	8 (3%)	0.669	7 (7%)	0.036	0 (0%)	0.001
19–25	35 (12%)	9 (10%)	0.199	10 (10%)	0.082	34 (13%)	0.669	15 (14%)	0.036	16 (8%)	0.001
26–45	183 (62%)	59 (66%)	0.199	68 (70%)	0.082	170 (63%)	0.669	64 (60%)	0.036	130 (66%)	0.001
46 and above	66 (23%)	21 (24%)	0.199	19 (20%)	0.082	60 (22%)	0.669	20 (19%)	0.036	52 (26%)	0.001
Gender											
Male	86 (29%)	33 (37%)	0.055	35 (36%)	0.075	76 (28%)	0.056	25 (24%)	0.103	36 (18%)	0.001
Female	207 (71%)	56 (63%)	0.055	62 (64%)	0.075	196 (72%)	0.056	81 (76%)	0.103	162 (82%)	0.001
Education level											
None	16 (5%)	4 (4%)	0.486	3 (3%)	0.557	15 (6%)	0.038	4 (4%)	0.136	12 (6%)	0.008
Primary	87 (30%)	31 (35%)	0.486	29 (30%)	0.557	84 (31%)	0.038	40 (38%)	0.136	59 (30%)	0.008
Secondary	103 (35%)	32 (36%)	0.486	33 (34%)	0.557	98 (36%)	0.038	33 (31%)	0.136	58 (29%)	0.008
Tertiary	87 (30%)	22 (25%)	0.486	32 (33%)	0.557	75 (28%)	0.038	29 (27%)	0.136	69 (35%)	0.008
Marital status											
Not married	95 (32%)	18 (20%)	0.003	17 (18%)	0.001	90 (33%)	0.381	37 (35%)	0.494	62 (31%)	0.558
Married	198 (68%)	71 (80%)	0.003	80 (82%)	0.001	182 (67%)	0.381	69 (65%)	0.494	136 (69%)	0.558
Network type											
Community	122 (42%)	17 (19%)	0.001	24 (25%)	0.001	112 (41%)	0.564	35 (33%)	0.024	84 (42%)	0.694
Naturally	171 (58%)	72 (81%)	0.001	73 (75%)	0.001	160 (59%)	0.564	71 (67%)	0.024	114 (58%)	0.694
	293	89		97		272		106		198	
*After stillbirth*											
Age group in years											
Less than 18	9 (3%)	0 (0%)	0.078	0 (0%)	0.109	9 (3%)	0.042	8 (5%)	0.030	0 (0%)	0.001
19–25	35 (12%)	9 (9%)	0.078	11 (11%)	0.109	35 (13%)	0.042	25 (15%)	0.030	13 (7%)	0.001
26–45	183 (62%)	69 (68%)	0.078	65 (63%)	0.109	172 (63%)	0.042	100 (61%)	0.030	133 (68%)	0.001
46 and above	66 (23%)	24 (24%)	0.078	27 (26%)	0.109	57 (21%)	0.042	32 (19%)	0.030	49 (25%)	0.001
Gender											
Male	86 (29%)	33 (32%)	0.410	39 (38%)	0.018	79 (29%)	0.566	44 (27%)	0.252	39 (20%)	0.001
Female	207 (71%)	69 (68%)	0.410	64 (62%)	0.018	194 (71%)	0.566	121 (73%)	0.252	156 (80%)	0.001
Education level											
None	16 (5%)	4 (4%)	0.398	2 (2%)	0.192	14 (5%)	0.238	8 (5%)	0.127	13 (7%)	0.078
Primary	87 (30%)	32 (31%)	0.398	29 (28%)	0.192	84 (31%)	0.238	56 (34%)	0.127	56 (29%)	0.078
Secondary	103 (35%)	31 (30%)	0.398	37 (36%)	0.192	97 (36%)	0.238	60 (36%)	0.127	61 (31%)	0.078
Tertiary	87 (30%)	35 (34%)	0.398	35 (34%)	0.192	78 (29%)	0.238	41 (25%)	0.127	65 (33%)	0.078
Marital status											
Not married	95 (32%)	23 (23%)	0.008	19 (18%)	0.001	89 (33%)	0.810	62 (38%)	0.032	53 (27%)	0.007
Married	198 (68%)	79 (77%)	0.008	84 (82%)	0.001	184 (67%)	0.810	103 (62%)	0.032	142 (73%)	0.007
Network type											
Community	122 (42%)	40 (39%)	0.539	33 (32%)	0.014	110 (40%)	0.084	60 (36%)	0.038	78 (40%)	0.422
Naturally	171 (58%)	62 (61%)	0.539	70 (68%)	0.014	163 (60%)	0.084	105 (64%)	0.038	117 (60%)	0.422
	293	102		103		273		165		195

## Social support and alter composition variables of personal networks

### Material support

According to alter characteristics, material support was reported to come from alters who were female 63% (*p* = 0.05), married 80% (*p* = 0.003), and from naturally occurring network members such as family and friends 81% (*p* = 0.001) during pregnancy. After experiencing a stillbirth, material support was reported to mainly come from alters who were 26–45 years 68% (*p* = 0.078) and were married 77% (*p* = 0.008).

### Financial support

Financial support was reported to come from alters who were married 82% (*p* = 0.001) and from naturally occurring network members 75% (*p* = 0.001) during pregnancy. After experiencing a stillbirth, material support was reported to mainly come from alters who were female 62% (*p* = 0.018) married 82% (*p* = 0.001), and were from naturally occurring network members 68% (*p* = 0.014).

### Emotional support

Emotional support was reported to come from alters who were male 72% (*p* = 0.05) and with a secondary level education, 36% (*p* = 0.03) during pregnancy and after experiencing a stillbirth emotional support was reported to come from respondents whose age ranged from 26–45 years 63% (*p* = 0.04).

### Instrumental support

During pregnancy, instrumental support was reported to come from alters aged 26–45 years 60% (*p* = 0.03) and from naturally occurring network members 67% (*p* = 0.02) while after experiencing a stillbirth instrumental support was reported to mainly come from alters who were aged 26–45 years 61% (*p* = 0.03), married 62% (*p* = 0.032) and from the naturally occurring network members 64% (*p* = 0.038).

### Information support

Information support to respondents during pregnancy was reported to come from network members who were females 66% (*p* = 0.001), between ages 26–45 years 82% (*p* = 0.001), and with a tertiary level education 35% (*p* = 0.008). On the other hand, after experiencing a stillbirth, information support was reported to come from alters who were in age category of 26–45 years 68% (*p* = 0.001), females 80% (*p* = 0.001), and who were married 73% (*p* = 0.007). Details are reflected in Table 3.

## Social support and network characteristics of personal networks

According to network characteristics, such as trust in the alter, emotional closeness with the alter, frequency of contact with the alter and count on alter for support were found to be significantly associated with all types of social support such as material, emotional, financial, instrumental and information support was reported to come from alters who were both during pregnancy and after experiencing a stillbirth as revealed in table.4*.*

**Table 4:** Bivariate association of social support with network characteristics

Variable	Total	Material	*P* value	Financial	*P* value	Emotional	*P* value	Instrumental	*P* value	Information	*P* value
During pregnancy											
Trust the alter											
Not at all	56 (19%)	7 (8%)	0.001	6 (6%)	0.001	46 (17%)	0.001	18 (17%)	0.001	20 (10%)	0.001
A little bit	157 (54%)	32 (36%)	0.001	42 (43%)	0.001	147 (54%)	0.001	43 (41%)	0.001	109 (55%)	0.001
Very much	80 (27%)	50 (56%)	0.001	49 (51%)	0.001	79 (29%)	0.001	45 (42%)	0.001	69 (35%)	0.001
Frequency of contact											
Once in 6 month	14 (5%)	3 (3%)	0.006	2 (2%)	0.003	14 (5%)	0.078	3 (3%)	0.001	8 (4%)	0.062
Once in 3 month	49 (17%)	5 (6%)	0.006	10 (10%)	0.003	41 (15%)	0.078	3 (3%)	0.001	25 (13%)	0.062
Once a month	69 (24%)	21 (24%)	0.006	20 (21%)	0.003	66 (24%)	0.078	23 (22%)	0.001	51 (26%)	0.062
Once a week	72 (25%)	24 (27%)	0.006	22 (23%)	0.003	67 (25%)	0.078	24 (23%)	0.001	50 (25%)	0.062
About every day	89 (30%)	36 (40%)	0.006	43 (44%)	0.003	84 (31%)	0.078	53 (50%)	0.001	64 (32%)	0.062
Emotional closeness											
Not at all	82 (28%)	6 (7%)	0.001	9 (9%)	0.001	67 (25%)	0.001	17 (16%)	0.001	40 (20%)	0.001
A little bit	155 (53%)	41 (46%)	0.001	49 (51%)	0.001	150 (55%)	0.001	51 (48%)	0.001	108 (55%)	0.001
Very much	56 (19%)	42 (47%)	0.001	39 (40%)	0.001	55 (20%)	0.001	38 (36%)	0.001	50 (25%)	0.001
Count on alter for support											
Not at all	98 (33%)	9 (10%)	0.001	10 (10%)	0.001	85 (31%)	0.016	22 (21%)	0.001	48 (24%)	0.001
A little bit	142 (48%)	35 (39%)	0.001	43 (44%)	0.001	136 (50%)	0.016	48 (45%)	0.001	105 (53%)	0.001
Very much	53 (18%)	45 (51%)	0.001	44 (45%)	0.001	51 (19%)	0.016	36 (34%)	0.001	45 (23%)	0.001
	293	89		97		272		106		198	
After stillbirth											
Trust the alter											
Not at all	56 (19%)	13 (13%)	0.001	11 (11%)	0.001	48 (18%)	0.041	31 (19%)	0.052	17 (9%)	0.001
A little bit	157 (54%)	41 (40%)	0.001	51 (50%)	0.001	148 (54%)	0.041	80 (48%)	0.052	108 (55%)	0.001
Very much	80 (27%)	48 (47%)	0.001	41 (40%)	0.001	77 (28%)	0.041	54 (33%)	0.052	70 (36%)	0.001
Frequency of contact											
Once in 6 month	14 (5%)	2 (2%)	0.001	1 (1%)	0.003	9 (3%)	0.001	2 (1%)	0.001	8 (4%)	0.054
Once in 3 month	49 (17%)	2 (2%)	0.001	11 (11%)	0.003	44 (16%)	0.001	17 (10%)	0.001	24 (12%)	0.054
Once a month	69 (24%)	23 (23%)	0.001	21 (20%)	0.003	63 (23%)	0.001	35 (21%)	0.001	49 (25%)	0.054
Once a week	72 (25%)	35 (34%)	0.001	36 (35%)	0.003	71 (26%)	0.001	42 (25%)	0.001	51 (26%)	0.054
About every day	89 (30%)	40 (39%)	0.001	34 (33%)	0.003	86 (32%)	0.001	69 (42%)	0.001	63 (32%)	0.054
Emotional closeness											
Not at all	82 (28%)	18 (18%)	0.001	16 (16%)	0.001	67 (25%)	0.001	35 (21%)	0.001	35 (18%)	0.001
A little bit	155 (53%)	39 (38%)	0.001	50 (49%)	0.001	152 (56%)	0.001	87 (53%)	0.001	108 (55%)	0.001
Very much	56 (19%)	45 (44%)	0.001	37 (36%)	0.001	54 (20%)	0.001	43 (26%)	0.001	52 (27%)	0.001
Count on alter for support											
Not at all	98 (33%)	17 (17%)	0.001	15 (15%)	0.001	85 (31%)	0.006	38 (23%)	0.001	48 (25%)	0.001
A little bit	142 (48%)	45 (44%)	0.001	52 (50%)	0.001	138 (51%)	0.006	85 (52%)	0.001	99 (51%)	0.001
Very much	53 (18%)	40 (39%)	0.001	36 (35%)	0.001	50 (18%)	0.006	42 (25%)	0.001	48 (25%)	0.001
	293	102		103		273		165		195	

## Predictors of social support

During pregnancy the predictors for provision of material support from the alters were being female (*p* = 0.05), married (*p* = 0.006), from naturally occurring network (*p* = 0.00), trusting the alter (*p* = 0.00), very much close emotionally (*p* = 0.00) and count on alter for support very much (*p* = 0.00). The predictors for provision of financial support from the alter were; being married (*p* = 0.001) from a naturally occurring network (*p* = 0.00), having much trust in the alter (*p* = 0.001), meeting alter at least once a week (*p* = 0.045), very close emotionally (*p* = 0.00) and counting on alter for support very much (*p* = 0.00). The predictors for provision of emotional support from the alter were having much trust in the alter (*p* = 0.049), being emotionally very close to the alter (*p* = 0.005), count on alter for support a little bit (*p* = 0.007). The predictors of instrumental support during pregnancy were; being aged 25 years and above (*p* = 0.049), being from a naturally occurring network (*p* = 0.029), meeting with the alter about every day (*p* = 0.010), very close to alter emotionally (*p* = 0.000), count on alter for support (*p* = 0.000). The predictors of information support during pregnancy were being 46 years and over (*p* = 0.000), female (*p* = 0.000), having much trust in the alter (*p* = 0.000), emotionally very close to the alter (*p* = 0.000) and very much counting on alter for support (*p* = 0.000). Details are reflected in Table 5.

**Table 5:** Predictors of social support during pregnancy

Variable	Material		Financial		Emotional		Instrumental		Information	
	c.PR (95% CI)	*P *value	c.PR (95% CI)	*P *value	c.PR (95% CI)	*P *value	c.PR (95% CI)	*P *value	c.PR (95% CI)	*P *value
*AltAge_1*										
18–25	1		1		1		1		1	
26–45	1.6 (0.85–2.93)	0.151	1.6 (0.92–2.91)	0.095	1 (0.9–1.05)	0.484	0.7 (0.49–1)	0.049	2 (1.31–2.92)	0.001
46 and above	1.6 (0.79–3.08)	0.205	1.3 (0.65–2.46)	0.486	1 (0.86–1.05)	0.339	0.6 (0.38–0.97)	0.037	2.2 (1.44–3.27)	0.000
*AltGender*										
Male	1		1		1		1		1	
Female	0.7 (0.5–1)	0.050	0.7 (0.53–1.02)	0.069	1.1 (0.99–1.16)	0.105	1.3 (0.93–1.95)	0.117	1.9 (1.44–2.42)	0.000
*AltEduc*										
None	1		1		1		1		1	
Primary	1.4 (0.58–3.49)	0.438	1.8 (0.61–5.15)	0.289	1 (0.9–1.18)	0.664	1.8 (0.76–4.43)	0.175	0.9 (0.66–1.24)	0.535
Secondary	1.2 (0.51–3.05)	0.635	1.7 (0.59–4.93)	0.322	1 (0.89–1.16)	0.829	1.3 (0.52–3.14)	0.587	0.8 (0.54–1.05)	0.089
Tertiary	1 (0.4–2.55)	0.981	2 (0.68–5.65)	0.212	0.9 (0.79–1.07)	0.280	1.3 (0.54–3.28)	0.531	1.1 (0.78–1.43)	0.718
*AltMarital*										
Not married	1		1		1		1		1	
Married	1.9 (1.2–2.99)	0.006	2.3 (1.42–3.59)	0.001	1 (0.91–1.03)	0.347	0.9 (0.65–1.23)	0.491	1.1 (0.88–1.25)	0.566
*NtwkType*										
Community role	1		1		1		1		1	
Naturally occuring	3 (1.88–4.86)	0.000	2.2 (1.46–3.23)	0.000	1 (0.95–1.09)	0.573	1.4 (1.04–2.02)	0.029	1 (0.83–1.14)	0.692
*TrustAlt*										
Not at all	1		1		1		1		1	
A little bit	1.1 (0.65–1.94)	0.672	1.7 (0.93–2.94)	0.087	1.1 (0.98–1.23)	0.102	0.9 (0.69–1.22)	0.564	2.3 (1.5–3.42)	0.000
Very much	2.6 (1.55–4.3)	0.000	2.6 (1.47–4.62)	0.001	1.1 (1–1.26)	0.049	1.2 (0.92–1.61)	0.166	2.9 (1.92–4.33)	0.000
*FreqCont*										
Once in six month	1		1		1		1		1	
Once in 3 month	0.3 (0.04–1.86)	0.189	3.1 (0.44–22.37)	0.253	1.4 (0.93–2.09)	0.104	2.4 (0.63–9.29)	0.195	0.9 (0.5–1.47)	0.574
Once a month	2.3 (0.62–8.81)	0.211	4.3 (0.62–29.22)	0.140	1.4 (0.95–2.11)	0.084	3.6 (0.96–13.11)	0.057	1.2 (0.77–2.01)	0.374
Once a week	3.4 (0.92–12.58)	0.066	7 (1.04–47.08)	0.045	1.5 (1.04–2.27)	0.032	4.1 (1.11–14.98)	0.034	1.2 (0.77–2)	0.379
About every day	3.1 (0.85–11.61)	0.085	5.3 (0.79–36.13)	0.085	1.5 (1.01–2.23)	0.042	5.4 (1.49–19.72)	0.010	1.2 (0.77–1.99)	0.376
*EmotCloss*										
Not at all	1		1		1		1		1	
somewhat	1.1 (0.7–1.87)	0.586	1.7 (1.01–2.72)	0.047	1.2 (1.08–1.33)	0.001	1.3 (0.99–1.75)	0.062	1.6 (1.24–2.14)	0.000
very close	3.7 (2.38–5.62)	0.000	3.4 (2.1–5.47)	0.000	1.2 (1.05–1.32)	0.005	1.8 (1.35–2.4)	0.000	2.2 (1.67–2.83)	0.000
*CountAltSupport*										
Not at all	1		1		1		1		1	
A little bit	1.8 (1.11–3)	0.017	2.4 (1.43–4)	0.001	1.1 (1.03–1.22)	0.007	1.5 (1.16–2.05)	0.003	1.4 (1.13–1.79)	0.003
Very much	4.4 (2.75–6.89)	0.000	4.4 (2.69–7.33)	0.000	1.1 (0.98–1.2)	0.106	2 (1.54–2.72)	0.000	1.8 (1.48–2.3)	0.000

After experiencing a stillbirth, the predictors for provision of material support were; alter being married (*p* = 0.020), from a naturally occurring network (*p* = 0.000) and among those alters whom respondents would count on for support (*p* = 0.019). For the provision of financial support after experiencing a stillbirth, the predictors were, being married (*p* = 0.004), from naturally occurring network (*p* = 0.000), and being counted on by the respondent to offer support whenever in need (*p* = 0.000). Regarding provision of emotional support the predictors were frequency of contact from once a week (*p* = 0.046) to about everyday (*p* = 0.058), being emotionally close from somewhat (*p* = 0.006) to very close (*p* = 0.015). The predictors for provision of instrumental support were being female (*p* = 0.029), coming from the naturally occurring networks (*p* = 0.013), having trust in the network member (*p* = 0.002), frequency of contact of about every day (*p* = 0.023) and ability to count on alter to provide support (*p* = 0.010). Lastly the predictors for provision of information support to mothers after experiencing a stillbirth were; alter being older than 46 years (*p* = 0.000), being female (*p* = 0.000), having a tertiary level of education (*p* = 0.028), from the naturally occurring network (*p* = 0.029) and having trust in the alter very much (*p* = 0.001). Details are reflected in Table 6.

**Table 6:** Predictors of social support after experiencing a stillbirth

Variable	Material		Financial		Emotional		Instrumental		Information	
	a.PR (95% CI)	*p* value	a.PR (95% CI)	*p* value	a.PR (95% CI)	*p* value	a.PR (95% CI)	*p* value	a.PR (95% CI)	*p* value
*AltAge_1*										
18–25	1		1		1		1		1	
26–45	1.3 (0.67–2.65)	0.412	1.1 (0.58–2.19)	0.725	1 (0.93–1.09)	0.812	0.7 (0.45–1.04)	0.078	1.9 (1.25–2.75)	0.002
46 and above	1.4 (0.69–3.03)	0.330	1 (0.45–2.19)	0.982	1 (0.9–1.07)	0.687	0.6 (0.36–1.03)	0.063	2.1 (1.42–3.12)	0.000
*AltGender*										
Male	1		1		1		1		1	
Female	0.9 (0.67–1.24)	0.573	1 (0.73–1.28)	0.803	1.1 (0.98–1.16)	0.145	1.5 (1.04–2.09)	0.029	2 (1.52–2.51)	0.000
*AltEduc*										
None	1		1		1		1		1	
Primary	1.2 (0.53–2.95)	0.613	1.3 (0.42–4.31)	0.615	1 (0.9–1.19)	0.636	1.8 (0.71–4.35)	0.225	1.1 (0.88–1.45)	0.335
Secondary	1 (0.43–2.46)	0.950	1.2 (0.38–3.92)	0.745	1 (0.89–1.17)	0.800	1.1 (0.44–2.86)	0.811	1 (0.78–1.34)	0.879
Tertiary	1.1 (0.43–2.59)	0.906	1.7 (0.54–5.31)	0.367	0.9 (0.79–1.09)	0.344	1.5 (0.6–3.97)	0.372	1.3 (1.03–1.76)	0.028
AltMarital										
Not married	1		1		1		1		1	
Married	1.9 (1.1–3.14)	0.020	2.2 (1.28–3.74)	0.004	1 (0.92–1.06)	0.753	1.1 (0.74–1.54)	0.737	1 (0.87–1.18)	0.862
*NtwkType*										
Community role	1		1		1		1		1	
Naturally occuring	3.2 (1.95–5.21)	0.000	2.5 (1.68–3.77)	0.000	1 (0.93–1.07)	0.928	1.5 (1.09–2.17)	0.013	1.2 (1.02–1.37)	0.029
*TrustAlt*										
Not at all	1		1		1		1		1	
A little bit	0.9 (0.46–1.76)	0.764	1 (0.46–2.07)	0.955	0.9 (0.81–1.09)	0.413	0.6 (0.39–0.81)	0.002	2.1 (1.31–3.37)	0.002
Very much	0.9 (0.42–2.12)	0.881	0.8 (0.32–1.87)	0.566	1 (0.82–1.11)	0.559	0.5 (0.34–0.81)	0.003	2.3 (1.4–3.88)	0.001
*FreqCont*										
Once in six month	1		1		1		1		1	
Once in 3 month	0.2 (0.04–1.42)	0.113	2.2 (0.33–14.92)	0.410	1.4 (0.94–2.01)	0.105	2.3 (0.61–8.51)	0.223	0.7 (0.46–1.03)	0.073
Once a month	1.4 (0.39–5.28)	0.593	2.4 (0.37–15.78)	0.352	1.4 (0.93–1.98)	0.116	3 (0.81–10.96)	0.099	0.9 (0.62–1.26)	0.492
Once a week	2.2 (0.59–7.97)	0.246	4 (0.62–26.19)	0.144	1.5 (1.01–2.1)	0.046	3.5 (0.97–12.89)	0.055	0.9 (0.61–1.23)	0.416
About every day	1.9 (0.52–7.07)	0.326	2.9 (0.45–18.9)	0.263	1.4 (0.99–2.08)	0.058	4.5 (1.23–16.1)	0.023	0.9 (0.63–1.24)	0.480
*EmotCloss*										
Not at all	1		1		1		1		1	
Somewhat	0.7 (0.37–1.33)	0.282	0.9 (0.47–1.9)	0.874	1.2 (1.05–1.37)	0.006	1.3 (0.9–1.96)	0.154	1.1 (0.77–1.48)	0.704
Very close	1.5 (0.69–3.16)	0.318	1.4 (0.6–3.09)	0.456	1.2 (1.04–1.43)	0.015	1.5 (0.86–2.51)	0.156	1.2 (0.85–1.75)	0.274
*CountAltSupport*										
Not at all	1		1		1		1		1	
A little bit	1.8 (1.06–3.19)	0.030	2.3 (1.32–4.14)	0.004	1 (0.94–1.08)	0.857	1.5 (1.09–2.04)	0.014	1.1 (0.83–1.38)	0.622
Very much	2.3 (1.15–4.77)	0.019	3.7 (1.79–7.61)	0.000	0.9 (0.82–1.06)	0.293	1.8 (1.16–2.94)	0.010	1.1 (0.81–1.49)	0.548

## Social support visualization at individual network level

**Fig. 2:**
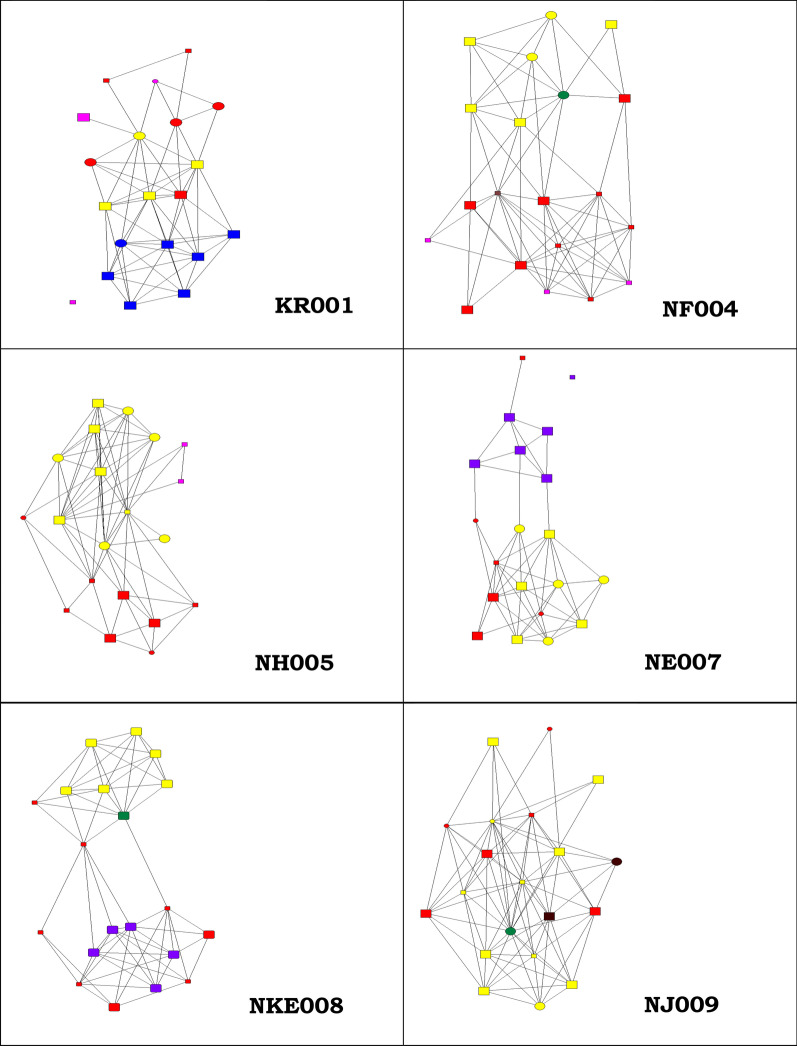
Social support visualization for six typical scenarios

Table 7 shows support as visualized through graphs at individual network level as perceived by the respondents. It reflects actors as nodes and relations connecting the actors as lines, it is reflected in the legend. Figure 2 shows typical scenarios which were picked from six respondents that reflected the different examples of actors and social support relational data. The visuals below represent social networks with health workers as integral (KR001, NF004, NH005) while (NF004, NKE008, NJ009,) shows a network with spousal support, (NF004, NH005, NJ009) reflecting a highly dense network and (KR001, NE007) shows crucial network members as isolates. Table 8 show results of the social support variables reported by each of the respondents per support type and the quality of relationship that existed between them and the alters.

**Table 7:** Legend for the social network graphs

Node shape: gender	Node size: support	Node colour: relationship
Circle: men	Small: less rely on alter for support	Spouse: green
Square: women	Large: count on alter for support	Family: yellow
		Friend: red
		Workmate: blue
		Health worker: pink
		Neighbor: black

**Table 8:** Social support after stillbirth descriptive statistics at ego level

Variable	Category	KR001 *N* (%)	NF004 *N* (%)	NH005 *N* (%)	NE007 *N* (%)	NK008 *N* (%)	NJ009 *N* (%)
Count on alter for support	Not at all	4 (20)	8 (40)	8 (40)	5 (25)	6 (30)	7 (35)
	A little bit	8 (40)	6 (30)	11 (55)	10 (50)	12 (60)	10 (50)
	Very much	8 (40)	6 (30)	1 (5)	5 (25)	2 (10)	3 (15)
Type of social support	Material (yes)	9 (45)	8 (40)	6 (30)	17 (85)	14 (70)	2 (10)
	Financial (yes)	4 (20)	9 (45)	8 (40)	18 (90)	17 (85)	3 (15)
	Emotional (yes)	17 (85)	19 (95)	20 (100)	20 (100)	19 (95)	20 (100)
	Instrumental (yes)	10 (50)	9 (45)	9 (45)	18 (90)	13 (65)	6 (30)
	Information (yes)	17 (85)	20 (100)	14 (70)	17 (85)	15 (75)	9 (45)
Frequency of contact	About every day	9 (45)	2 (10)	4 (20)	6 (30)	3 (15)	6 (30)
	Once a week	3 (15)	13 (65)	8 (40)	6 (30)	11 (55)	4 (20)
	Once a month	4 (20)	3 (15)	7 (35)	7 (35)	4 (20)	5 (25)
	Once in 3 months	2 (10)	2 (10)	1 (5)	1 (5)	1 (5)	5 (25)
	Once in 6 months	2 (10)	–	–	–	1 (5)	–
Emotional closeness	Not at all	6 (30)	2 (10)	10 (50)	5 (25)	6 (30)	4 (20)
	Somewhat close	7 (35)	11 (55)	8 (40)	9 (45)	12 (60)	11 (55)
	Very close	7 (35)	7 (35)	2 (10)	6 (30)	2 (10)	5 (25)
How alter got to know about stillbirth news	Told them myself	8 (40)	9 (45)	5 (25)	3 (15)	3 (15)	4 (20)
	Knew by themselves	2 (10)	1 (5)	2 (10)	1 (5)	6 (30)	6 (30)
	Through network members	7 (35)	10 (50)	13 (65)	16 (80)	11 (55)	10 (50)
	Other means	3 (15)	–	–	–	–	–
Change in friendship after stillbirth	As before	11 (55)	11 (55)	11 (55)	7 (35)	13 (65)	18 (90)
	Better now	8 (40)	9 (45)	8 (40)	13 (65)	7 (35)	2 (10)
	Worse than before	1 (5)	–	1 (5)	–	–	–

Table 9 reports the structure of the network and overall the *Degree centrality* which denotes tie strength as reflected in the networks with most direct ties shows that of the six cases presented, the degree centrality ranged from 5.3, 6.1, 6.3, 6.8 and was highest in networks with spousal support 6.9 (NF004) and 8.05 (NJ009).

**Table 9:** Structural measures of personal networks for six scenarios

	Variable	KR001	NF004	NH005	NE007	NK008	NJ009
1	Density	0.32	0.36	0.35	0.27	0.33	0.42
2	Degree centrality (mean)	6.1	6.9	6.8	5.3	6.3	8.05
3	Closeness centrality (mean)	41.3	33.6	34.9	47.8	38.1	30.5
4	Betweenness centrality (mean)	7.3	7.3	7.95	9.65	9.55	11.5
5	Number of ties	122	138	136	106	126	161
6	Isolates	1	0	0	1	0	0

Results from the *closeness centrality* which refers to a relationship traversing fewer degrees of separation or closest to other nodes in the network shows that the mean closeness centrality score of all the six cases presented here ranged from 30.05 to 47.8 and was highest among respondent’s networks with workmates (KR001, NE007).

*Betweenness centrality* which denotes the extent to which a node lies on the shortest paths between other nodes on the main information pathway had results revealing that the mean scores were lowest among respondents that had health workers as integral actors in their networks (KR001, NF004 and NH005).

## Discussion

This study set out to explore the role and attributes of network members in provision of social support to pregnant mothers before and after experiencing a stillbirth. This is particularly important because whereas previous studies have investigated the role of social support among pregnant women, the outcomes of focus were on maternal wellbeing postpartum in general and not necessarily because they had experienced a stillbirth [28]. Even where the focus seemed to be on pregnancy outcomes, having a stillbirth was not among those assessed [38]. Overall, results revealed availability of social support from a variety of network members. Most frequently mentioned form of support was intangible support which included emotional and information support which came from mainly females who were married. The network quality characteristics such as trust, frequency of contact and alters that were counted on to provide support to mothers were observed to be influence a great deal of social support.

### Availability of social support

The findings revealed that social support was available from all network relations mentioned by the respondents. No major variations were observed between the two time periods during pregnancy and after experiencing a stillbirth. During pregnancy only 10 (3.4%) relations (network member mentioned) were noted not to have provided any form of support while 9 (3.1%) were noted for the same after experiencing a stillbirth. All forms of support from network members increased slightly after experiencing a stillbirth compared to the same during pregnancy save for information support which reduced. This is expected since communities tend to empathize with grieving families and as a result will tend to offer any possible support available at that particular time. Another possible explanation could be that given the alters elicited during the study were perceived to have an already existing relationship with the respondent, provision of such support would be expected and forthcoming. Targeting mothers immediately to engage them in postnatal counseling is key to guide them in subsequent pregnancies since studies have long established that the desire for more children increases with a loss of a child and women tend to conceive immediately [54]. Purposing the support at that point to one which will help the mother recover from the situation empowered is important. Such support can range from information and emotional since many women would wish to be guided through their post-partum healthcare seeking and emotional recovery.

The most commonly available support that respondents received was in form of intangible support such as emotional and information support. It was reported from 96% of the alters throughout pregnancy and after experiencing a stillbirth. Similar results have been reported elsewhere [4, 39] where emotional support was found to be the most prevalent among network members. It provides a sense of empathy, accompaniment and understanding [40]. This demonstrates that the quality of information shared with a pregnant mother or even after experiencing a stillbirth through emotional support is crucial. Ensuring that quality information is offered in the context of averting stillbirth risk factors is key. One reason as to why emotional and information support was more prevalent could be that it is readily available unlike other forms of support such as material [41]. However, information support reduced slightly after experiencing a stillbirth most likely a reflection of a sense of loss and fear of causing more distress and stigma to the affected mother. It may also have been due to a lack of confidence on which information to provide then that would be beneficial to the grieving mother and her family. Mapping of network members likely to offer this kind of support and empower them through programs identifying community peer supporters is one way to achieve this.

### Alter characteristics associated with social support

Most of the social support reportedly received by the respondents came from females who were married. These variables highlight the importance of gender and social status such as marital status in predicting social support both during pregnancy and after experiencing a stillbirth. The results add to the existing body of knowledge that highlighted the importance of gender in provision of social support [42]. Elsewhere studies have also identified marital status as being key in predicting provision of social support [43]. Regarding the relationships between the ego and alter, our results show that much of the social support come from alters who were from the naturally occurring networks such as family and friends. This observation builds on the existing literature where studies have previously reported the important contribution of family and friends in the provision of social support whenever in need [44]. As previously reported, the higher proportion of family members within one’s network might be a reflection of the group orientation around family which is typical in many sub-Sahara African settings including Uganda [45]. Also this may serve to emphasize the role of family as a major factor in decision making dynamics surrounding maternal healthcare and overall health in general [46, 47]. For friends, they are an immediate point of reference whenever faced with life threatening challenges including negative maternal health outcomes. They serve to cushion pregnant women from adverse life events and also help uplift them after experiencing such [48]. Interventions aiming at addressing stillbirth reduction therefore ought not to view pregnant women in isolation of the surrounding environment especially their embedded relationships.

## Social network relational characteristics associated with social support

From our study, we observed that social support tended to follow through certain patterns of network relational characteristics. Trust, frequency of contact and alters that were counted on for support were more likely to provide the same. Trust builds confidence within individuals in a particular relation and will tend to seek or receive support among those they feel comfortable with and have certain level of trust [49]. Frequency of contact has a mutually reinforcing interaction. People in such relationships have a particular pattern of interaction which informs who they are likely to turn to for support whenever in need [50]. Count on alter for support has a re-assuring effect which may make support seekers alter behaviours to seek support from people they are assured of getting it from. Therefore when considering social support in terms of the quality of relations, it is important to note that trust in the alter, frequency of contact and perception of ability to provide support are important in predicting the actual support hence need to be considered when designing community level interventions to address stillbirth risks.

## Social network structure characteristics

Previous studies have observed the role of network structure in predicting certain health outcomes such as transmission patterns of infectious diseases and sexual behaviours in networks [51] or contagion of health harming behaviors and outcomes within a network such as obesity [52], alcoholism and drug abuse [53]. Another important finding from our study is that in terms of network structure, the mean degree centrality scores ranged from 5.3 to 8.05 and were highest among networks with spousal support. This means that spouses play a critical role not only in providing but also galvanizing support among network members. Closeness centrality scores revealed being dominant among women with networks that had co-workers as major actors. The underlying assumption behind this could be that co-workers may act other roles within the same network and thereby bridging the distance among other network actors such as friends, neighbors and family members. It may also be a reflection that the more women have many co-workers as part of their networks, the more such networks become closely knit. The mean betweenness centrality scores revealed that it was highest where the network contained health workers as nodes within the pregnant woman’s network. In our case it may have facilitated the shortening of the distance between nodes especially with regards to maternal health related information. Lastly, as established elsewhere, a higher betweenness can be important in presenting quality social support to the ego through linking with other indirect relationships [40].

## Limitations

One limitation to this study as it relates with social network studies is the likelihood of over estimating or underreporting social support given the prevailing circumstances. In this case, mothers that perceive to have received less of the expected support tend to under report. Besides, participants were recruited from public and PNFP facilities whose clients share similar characteristics such as a preference for free or less costly maternal health care services. Therefore the results cannot be generalized to the whole population including mothers who seek care from private for profit facilities. They are perceived as being capable of meeting the healthcare costs and may deliberately not seek some form of support from their network peers such as financial and material support. However during design and data collection, efforts were made to recruit mothers from facilities at different levels of service provision for both public and PNFP. Still, the results, discussion and conclusions are drawn from a study conducted in a single district with characteristics of a peri-urban livelihood context. Therefore, caution is called for when generalizing these results outside the study area with varying social-cultural and economic contexts that are central to social network functioning and maternal healthcare support seeking. Lastly, this was a cross sectional network survey and thus results may not be used to assess social support over time during pregnancy and after experiencing a stillbirth. We recommend a larger study covering more districts with varying characteristics that incorporate private for profit maternal health service users with a qualitative component to explore in-depth social support dynamics among mothers.

## Conclusions

A great potential for social support exists within women’s social networks to help address stillbirth risk factors during pregnancy and cope after experiencing the same. Alter characteristics like being female, married, and from naturally occurring networks together with relational characteristics such as trust, frequency of contact, and count on alter for support were predictors of eventual social support. Interventions aiming at addressing stillbirth risks at the community level ought to harness these network characteristics for benefits to the mothers.

## Supplementary Information

**Additional file 1**. Social network analysis questionaire.

## Data Availability

The datasets used and/or analyzed during the current study are available from the corresponding author on reasonable request.

## Abbreviations

ANC: Antenatal Care
CHEW: Community Health Extension Workers
EmOC: Emergency Obstetric Care
ENAP: Every Newborn Action Plan
GFF: Global Financing Facility
HC: Health Centre
HSDP: Health Sector Development Plan
HIV: Human Immunodeficiency Virus
PMTCT: Prevention of Mother to Child Transmission.
PNFP: Private Not for Profit
SNA: Social Network Analysis

## References

[1] Tani F, Castagna V (2017). Maternal social support, quality of birth experience, and post-partum depression in primiparous women. J Matern Fetal Neonatal Med.

[2] Kritsotakis G, Vassilaki M, Melaki V, Georgiou V, Philalithis AE, Bitsios P (2013). Social capital in pregnancy and postpartum depressive symptoms: a prospective mother–child cohort study (the Rhea study). Int J Nurs Stud.

[3] Hetherington E, McDonald S, Williamson T, Patten SB, Tough SC (2018). Social support and maternal mental health at 4 months and 1 year postpartum: analysis from the All Our Families cohort. J Epidemiol Community Health.

[4] Abdollahpour S, Ramezani S, Khosravi A. Perceived social support among family in pregnant women. 2015.

[5] Logsdon MC, Koniak-Griffin D (2005). Social support in postpartum adolescents: guidelines for nursing assessments and interventions. J Obstet Gynecol Neonatal Nurs.

[6] Shakya HB, Stafford D, Hughes DA, Keegan T, Negron R, Broome J (2017). Exploiting social influence to magnify population-level behaviour change in maternal and child health: study protocol for a randomised controlled trial of network targeting algorithms in rural Honduras. BMJ Open.

[7] Lin S, Faust L, Robles-Granda P, Kajdanowicz T, Chawla NV (2019). Social network structure is predictive of health and wellness. PLoS ONE.

[8] Valente TW (2012). Network interventions. Science.

[9] Igumbor JO, Ouma J, Otwombe K, Musenge E, Anyanwu FC, Basera T (2019). Effect of a Mentor Mother Programme on retention of mother-baby pairs in HIV care: a secondary analysis of programme data in Uganda. PLoS ONE.

[10] Muheirwe F, Nuhu S (2019). Men’s participation in maternal and child health care in Western Uganda: perspectives from the community. BMC Public Health.

[11] Kakaire O, Kaye DK, Osinde MO (2011). Male involvement in birth preparedness and complication readiness for emergency obstetric referrals in rural Uganda. Reprod Health.

[12] Namujju J, Muhindo R, Mselle LT, Waiswa P, Nankumbi J, Muwanguzi P (2018). Childbirth experiences and their derived meaning: a qualitative study among postnatal mothers in Mbale regional referral hospital, Uganda. Reprod Health.

[13] Patil CL, Abrams ET, Klima C, Kaponda CP, Leshabari SC, Vonderheid SC (2013). CenteringPregnancy-Africa: a pilot of group antenatal care to address Millennium Development Goals. Midwifery.

[14] Sharma J, O’Connor M, Jolivet RR (2018). Group antenatal care models in low-and middle-income countries: a systematic evidence synthesis. Reprod Health.

[15] Ekirapa-Kiracho E, Paina L, Muhumuza Kananura R, Mutebi A, Jane P, Tumuhairwe J (2017). ‘Nurture the sprouting bud; do not uproot it’. Using saving groups to save for maternal and newborn health: lessons from rural Eastern Uganda. Glob Health Action.

[16] Mutebi A, Muhumuza Kananura R, Ekirapa-Kiracho E, Bua J, Namusoke Kiwanuka S, Nammazi G (2017). Characteristics of community savings groups in rural Eastern Uganda: opportunities for improving access to maternal health services. Glob Health Action.

[17] Ayiasi RM, Atuyambe LM, Kiguli J, Orach CG, Kolsteren P, Criel B (2015). Use of mobile phone consultations during home visits by Community Health Workers for maternal and newborn care: community experiences from Masindi and Kiryandongo districts, Uganda. BMC Public Health.

[18] Rudrum S (2016). Traditional birth attendants in rural northern Uganda: policy, practice, and ethics. Health Care Women Int.

[19] Bua J, Paina L, Kiracho EE (2015). Lessons learnt during the process of setup and implementation of the voucher scheme in Eastern Uganda: a mixed methods study. Implement Sci.

[20] Health Mo. Health Sector Development Plan 2015/16–2019/20. 2015.

[21] Organization WH. Every newborn: an action plan to end preventable deaths. 2014.

[22] Health Mo. Annual Health Sector Performance Report 2018/2019. 2019.

[23] Frøen JF, Friberg IK, Lawn JE, Bhutta ZA, Pattinson RC, Allanson ER (2016). Stillbirths: progress and unfinished business. The Lancet.

[24] De Bernis L, Kinney MV, Stones W, ten Hoope-Bender P, Vivio D, Leisher SH (2016). Stillbirths: ending preventable deaths by 2030. The Lancet.

[25] Kiguli J, Namusoko S, Kerber K, Peterson S, Waiswa P (2015). Weeping in silence: community experiences of stillbirths in rural eastern Uganda. Glob Health Action.

[26] Kiguli J, Munabi IG, Ssegujja E, Nabaliisa J, Kabonesa C, Kiguli S (2016). Stillbirths in sub-Saharan Africa: unspoken grief. The Lancet.

[27] George A, Young M, Bang A, Chan KY, Rudan I, Victora CG (2011). Setting implementation research priorities to reduce preterm births and stillbirths at the community level. PLoS Med.

[28] Balaji AB, Claussen AH, Smith DC, Visser SN, Morales MJ, Perou R (2007). Social support networks and maternal mental health and well-being. J Womens Health.

[29] Edmonds JK, Hruschka D, Bernard HR, Sibley L (2012). Women’s social networks and birth attendant decisions: application of the network-episode model. Soc Sci Med.

[30] Mukong AK, Burns J. Social networks and maternal health care utilisation in Tanzania. 2015.

[31] Ssali S, Wagner G, Tumwine C, Nannungi A, Green H. HIV clients as agents for prevention: a social network solution. AIDS research and treatment. 2012;2012. 10.1155/2012/815823 PMC3361150 22666563

[32] Green HD, Atuyambe L, Ssali S, Ryan GW, Wagner GJ (2011). Social networks of PLHA in Uganda: implications for mobilizing PLHA as agents for prevention. AIDS Behav.

[33] Ssegujja E, Ddumba I, Andipartin M (2021). Prioritization of interventions in pursuit of maternal health policy objectives to mitigate stillbirth risks. An exploratory qualitative study at subnational level in Uganda. BMC Health Serv Res.

[34] Pustejovsky JE, Spillane JP (2009). Question-order effects in social network name generators. Soc Netw.

[35] Burt RS (2000). The network structure of social capital. Res Org Behav.

[36] Campbell KE, Lee BA (1991). Name generators in surveys of personal networks. Soc Netw.

[37] De la Haye K, Green HD, Kennedy DP, Zhou A, Golinelli D, Wenzel SL (2012). Who is supporting homeless youth? Predictors of support in personal networks. J Res Adolesc.

[38] Elsenbruch S, Benson S, Rücke M, Rose M, Dudenhausen J, Pincus-Knackstedt MK (2007). Social support during pregnancy: effects on maternal depressive symptoms, smoking and pregnancy outcome. Hum Reprod.

[39] Fernández-Peña R, Molina JL, Valero O (2018). Personal network analysis in the study of social support: the case of chronic pain. Int J Environ Res Public Health.

[40] Fernández-Peña R, Molina JL, Valero O (2020). Satisfaction with social support received from social relationships in cases of chronic pain: the influence of personal network characteristics in terms of structure, composition and functional content. Int J Environ Res Public Health.

[41] Hood S, Golembiewski E, Benbow K, Sow H, Sanders TV (2017). Who can I turn to? Emotional support availability in African American social networks. Social Sciences.

[42] McGuire GM (2012). Race, gender, and social support: A study of networks in a financial services organization. Sociol Focus.

[43] Kalmijn M, Vermunt JK (2007). Homogeneity of social networks by age and marital status: a multilevel analysis of ego-centered networks. Soc Netw.

[44] Benkel I, Wijk H, Molander U (2009). Family and friends provide most social support for the bereaved. Palliat Med.

[45] Green HD, Tucker JS, Golinelli D, Wenzel SL (2013). Social networks, time homeless, and social support: a study of men on Skid Row. Netw Sci (Camb Univ Press).

[46] Zhang AY, Siminoff LA, editors. The role of the family in treatment decision making by patients with cancer. Oncology nursing forum; 2003. Oncology Nursing Society. 10.1188/03.ONF.1022-1028 14603359

[47] Ganle JK, Obeng B, Segbefia AY, Mwinyuri V, Yeboah JY, Baatiema L (2015). How intra-familial decision-making affects women’s access to, and use of maternal healthcare services in Ghana: a qualitative study. BMC Pregnancy Childbirth.

[48] Arora NK, Finney Rutten LJ, Gustafson DH, Moser R, Hawkins RP (2007). Perceived helpfulness and impact of social support provided by family, friends, and health care providers to women newly diagnosed with breast cancer. Psychooncology.

[49] Mortenson ST (2009). Interpersonal trust and social skill in seeking social support among Chinese and Americans. Commun Res.

[50] Al-Kandari YY (2011). Relationship of strength of social support and frequency of social contact with hypertension and general health status among older adults in the mobile care unit in Kuwait. J Cross Cult Gerontol.

[51] Smith A, Grierson J, Wain D, Pitts M, Pattison P (2004). Associations between the sexual behaviour of men who have sex with men and the structure and composition of their social networks. Sexually transmitted infections.

[52] Cohen-Cole E, Fletcher JM (2008). Is obesity contagious? Social networks vs. environmental factors in the obesity epidemic. J Health Econ.

[53] Chan GH, Lo TW, Lee GK-W, Tam CH-L (2020). Social capital and social networks of hidden drug abuse in Hong Kong. Int J Environ Res Public Health.

[54] Murphy M, Savage E, O'Donoghue K, Leary JO, Leahy-Warren P. Trying to conceive: An interpretive phenomenological analysis of couples' experiences of pregnancy after stillbirth. Women Birth 2020:S1871–5192(20)30373-5. 10.1016/j.wombi.2020.10.016 10.1016/j.wombi.2020.10.016 33176997

