# Supplementary material for: The role and attributes of social networks in the provision of support to women after stillbirth: experiences from Uganda
Source: BMC Womens Health. 2021 Oct 6;21:352. doi: 10.1186/s12905-021-01498-9 (PMC8496046; doi:10.1186/s12905-021-01498-9)
Supplement: Supplementary file 1 — Additional file 1. Social network analysis questionaire. [file 12905_2021_1498_MOESM1_ESM.docx]

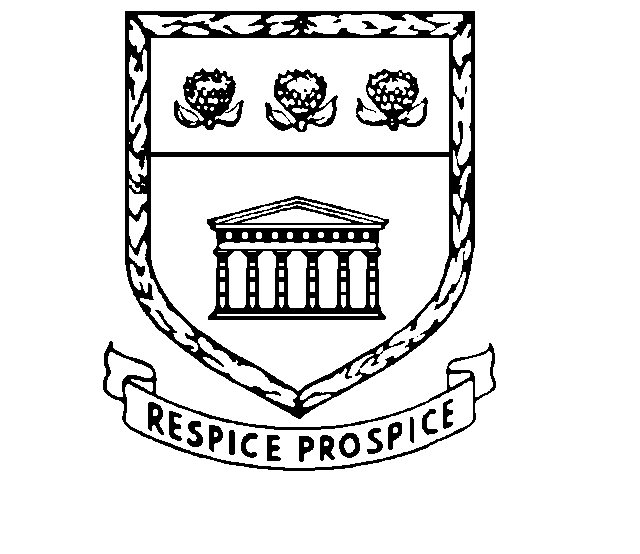


**
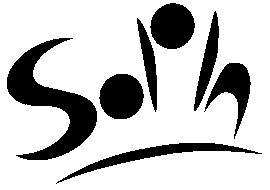
UNIVERSITY OF THE WESTERN CAPE**

**School of Public Health**

Private Bag X17 ● **BELLVILLE** ● 7535 ● South Africa

Tel: 021- 959 2809, Fax: 021- 959 2872

**E-mail:** [**ssega2001@yahoo.com**](mailto:ssega2001@yahoo.com)**,** [**bvanwyk@uwc.ac.za**](mailto:bvanwyk@uwc.ac.za)

**Introduction**

This study is being conducted as part of a PhD in Public Health, as a requirement leading to a thesis for examination. The study will focus on your perceptions of stillbirth risks and the role of women’s social networks in providing coping mechanisms to addressing the perceived risks. If you agree to participate, completing the social network analysis survey will take about [1 hour]. The survey will be administered by a research team member and will be provided to each participant individually. The questions in the survey include your social demographic characteristics, information about your network characteristics such as composition, size, how you help each other, whether network members are from formal institutions like health facilities, information flow, support direction and density as well as the functional aspects of the network such as the nature of support received to address stillbirth risk factors. It is your decision whether or not to be in this study. You can stop your participation in the survey at any time or you can choose not to respond to specific questions in the survey but continue with the rest.

| **s/n** | **Question** | **Response** | **Code** |
| --- | --- | --- | --- |
| 100 | ***Respondent characteristics*** |  |  |
| 101 | Age |  |  |
| 102 | Education | 1. No education 2. Primary level 3. Some secondary 4. Completed secondary 5. Tertially |  |
| 103 | Religious affiliation | 1. Catholic 2. Protestant 3. Muslim 4. Pentecostal 5. Traditionist 6. Non believer 7. Other |  |
| 104 | Marital status | 1. Married 2. Not married |  |
| 105 | What category would best describe your current employment | 1. Housewife 2. Peasant farmer 3. Casual laborer 4. Salaried worker 5. Petty trader 6. Other |  |
|  | ***Household characteristics*** |  |  |
| 106 | Number of people living in household |  |  |
| 107 | Number of children below 10 years living in the household |  |  |
| 108 | Family type | 1. Nuclear 2. Extended |  |
| 109 | Estimated Household income in a month |  |  |
|  | ***Respondents maternal health characteristics*** |  |  |
| 110 | Age at first pregnancy (in complete years) |  |  |
| 111 | Age at first live birth (in complete years) |  |  |
|  | ***Pregnancy history*** |  |  |
| 112 | Number of total pregnancies |  |  |
| 113 | Number of live birth |  |  |
| 114 | Ever experienced pregnancy complications | 1. Yes 2. No |  |
| 115 | Number of premature birth |  |  |
| 116 | Number of miscarriages |  |  |
| 117 | Number of voluntary abortions |  |  |
| 118 | Outcome of the last pregnancy | 1. Live born 2. Stillbirth 3. Abortion 4. Miscarriage |  |
| 119 | Number of living children |  |  |
| 120 | Current status of newborn: Alive | 1. Yes 2. No |  |
| 121 | Sex of the newborn | 1. Male 2. Female |  |
|  | ***Obstetric history*** |  |  |
| 122 | Number of vaginal births |  |  |
| 123 | Number of caesarian sections |  |  |
| 124 | Reason for caesarian section |  |  |
| 125 | Number of facility deliveries |  |  |
| 126 | Mode of past delivery | 1. Spontaneous vaginal delivery 2. Assisted vaginal delivery 3. Non elective caesarian section 4. Elective caesarian section |  |
|  | ***Fertility intentions/desires*** |  |  |
| 127 | Current use of family planning | 1. Yes 2. No |  |
| 128 | Number of children desired |  |  |
| 129 | Do you plan on having more children? | 1. Yes 2. No |  |
| 130 | How many more do you intend to have | 1. one 2. two 3. three 4. four and more |  |
| 131 | When do you intend to have the child | 1. next year 2. after two years 3. after five years 4. 6yrs and above |  |
| 200 | ***Name atleast 20 network members of 18 years and above with whom you have had contact in the past 6 months and whom you interacted with during you last pregnancy (1------------------20)*** |  |  |
|  | 1 |  |  |
|  | 2 |  |  |
|  | 3 |  |  |
|  | 4 |  |  |
|  | 5 |  |  |
|  | 6 |  |  |
|  | 7 |  |  |
|  | 8 |  |  |
|  | 9 |  |  |
|  | 10 |  |  |
|  | 11 |  |  |
|  | 12 |  |  |
|  | 13 |  |  |
|  | 14 |  |  |
|  | 15 |  |  |
|  | 16 |  |  |
|  | 17 |  |  |
|  | 18 |  |  |
|  | 19 |  |  |
|  | 20 |  |  |
| 300 | ***Description of the network members [for each of the network member listed above answer the following questions]*** |  |  |
| 301 | What is the age of [network member] |  |  |
| 302 | What is the gender of [network member] | 1. Male 2. Female |  |
| 303 | What is the education level of [network member] | 1. No education 2. Primary 3. Some secondary 4. Completed secondary 5. Tertiary |  |
| 304 | Marital status of the [network member] | 1. Married 2. Not married |  |
| 305 | What is [network member$$$] relationship to you | 1. Sexual partner Family 2. Friend 3. Neighbor 4. Health Service provider 5. Community leader |  |
| 306 | What type of family member is [network member$$$] | 1. Grand parent 2. Parent 3. Sibling 4. Aunt/uncle 5. Cousin 6. Other |  |
| 307 | How much do you trust [network member$$$] | 1. Not at all 2. A little bit 3. Very much |  |
| 308 | How often do you have contact with [network member$$$] | 1. Never 2. One time a month 3. One time a week 4. Several times a week 5. About every day |  |
| 309 | How close are you with [network member$$$] emotionally | 1. Not at all 2. Somewhat 3. Very close |  |
| 310 | How often do you ask for advice from [network member] when you have a problem | 1. Never 2. Somewhat 3. Often |  |
| 311 | I can count on [network member] to give me the support I need no matter what | 1. Not at all 2. A little bit 3. Very much |  |
| 400 | ***Social support characterization[for each of the network member listed above answer the following questions]*** |  |  |
| 401 | How often have you provided [network member] with material/tangible support such as food or clothes when needed | 1. Not at all 2. A little bit 3. Very much |  |
| 402 | How often did [network member] provide you with material/tangible support such as food or clothes because you were pregnant | 1. Not at all 2. A little bit 3. Very much |  |
| 403 | How often have you provided [network member] with financial support when needed | 1. Not at all 2. A little bit 3. Very much |  |
| 404 | How often have you asked [network member] for Financial support (to buy medicine, go to health centre) | 1. Not at all 2. A little bit 3. Very much |  |
| 405 | How often did you Financial support from [network member] (to buy medicine, go to health centre) | 1. Not at all 2. A little bit 3. Very much |  |
| 406 | How often have you provided [network member] with Emotional support when needed (have a good time with, understands your problems, to love and make you feel wanted) | 1. Not at all 2. A little bit 3. Very much |  |
| 407 | How often did [network member] provide you with Emotional support because you were pregnant(have a good time with, understands your problems, to love and make you feel wanted) | 1. Not at all 2. A little bit 3. Very much |  |
| 408 | How often have you provided [network member] with Instrumental support (help out with domestic chores, to prepare you meals when unable, escort to facility) when needed | 1. Not at all 2. A little bit 3. Very much |  |
| 409 | How often did [network member] provide you with Instrumental support (help out with domestic chores, to prepare you meals when unable, escort to facility) because you were pregnant | 1. Not at all 2. A little bit 3. Very much |  |
| 410 | How often did you ask [network member] for advice or information about pregnancy related health such as (to run to for suggestion to deal with pregnancy complication, listen to you when need someone to talk to about pregnancy, to give you advise about pregnancy health, to give you information to help you understand a situation, someone to confide in, to share your most private pregnancy worries) in the past six months | 1. Not at all 2. A little bit 3. Very much |  |
| 411 | How often did [network member] talk to you and give you advice about pregnancy related health such as (to run to for suggestion to deal with pregnancy complication, listen to you when need someone to talk to about pregnancy, to give you advise about pregnancy health, to give you information to help you understand a situation, someone to confide in, to share your most private pregnancy worries) during your last pregnancy | 1. Not at all 2. A little bit 3. Very much |  |
| 412 | How often did you provide [network member] with positive social interaction (to have a good time with, to get together with for relaxation, to do something enjoyable with) in the past six months | 1. Not at all 2. A little bit 3. Very much |  |
| 413 | How often did [network member] make you have a positive social interaction (to have a good time with, to get together with for relaxation, to do something enjoyable with) during your last pregnancy | 1. Not at all 2. A little bit 3. Very much |  |
| 414 | How often did you make [network member] feel affectionate (to love and make you feel wanted, who shows you love and affection, who hugs you) in the past six months | 1. Not at all 2. A little bit 3. Very much |  |
| 415 | How often did [network member] make you feel affectionate (to love and make you feel wanted, who shows you love and affection, who hugs you) during your last pregnancy | 1. Not at all 2. A little bit 3. Very much |  |
| 500 | ***How do the network members relate to one another [for all network members mentioned show how they relate with one another]*** |  |  |
| 501 | Does [network member$$$] know [network member&&&] | 1. Yes 2. No 3. Don’t know |  |
| 502 | How frequently would you say [network member$$$] and [network member&&&] have had contact with each other in the past 6 months, either face-to-face or by phone? | 1. Not at all 2. A little bit 3. Very much |  |
